# Nanobiocomposite Polymer as a Filter Nanosponge for Wastewater Treatment

**DOI:** 10.3390/molecules26133992

**Published:** 2021-06-30

**Authors:** Anny Leudjo Taka, Elvis Fosso-Kankeu, Xavier Yangkou Mbianda, Michael Klink, Eliazer Bobby Naidoo

**Affiliations:** 1 Department of Chemistry/Biotechnology, Vaal University of Technology, Vanderbijlpark 1900, South Africa; michaelk1@vut.ac.za (M.K.); bobbyvut@gmail.com (E.B.N.); 2 Institute of Chemical & Biotechnology, Vaal University of Technology, Southern Gauteng Science and Technology Park, Sebokeng 1983, South Africa; 3 Department of Electrical and Mining Engineering, College of Science Engineering and Technology, Florida Science Campus, University of South Africa, Roodepoort 1790, South Africa; elvisfosso.ef@gmail.com; 4 Department of Chemical Sciences, University of Johannesburg, Doornfontein, Johannesburg 2028, South Africa

**Keywords:** nanobiocomposite, nanosponge, polymer, water treatment

## Abstract

A multifunctional nanobiocomposite polymer was developed in this study through a cross-linking polymerization of cyclodextrin with phosphorylated multi-walled carbon nanotubes followed by sol-gel to incorporate TiO_2_ and Ag nanoparticles. This work’s novelty was to prove that the developed nanobiocomposite polymer is a potential filter nanosponge capable of removing organic, inorganic, and microorganisms’ pollutants from wastewater samples. The synthesized multifunctional nanobiocomposite polymer was characterized using a range of spectroscopy and electron microscopy techniques. Fourier-transform infrared (FTIR) confirmed the presence of oxygen-containing groups on the developed nanobiocomposite polymer and carbamate linkage (NH(CO)) distinctive peak at around 1645 cm^−1^, which is evidence that the polymerization reaction was successful. The scanning electron microscopy (SEM) image shows that the developed nanobiocomposite polymer has a rough surface. The Dubinin–Radushkevich and the pseudo-second-order kinetic models best described the adsorption mechanism of Co^2+^ and TCE’s onto pMWCNT/CD/TiO_2_-Ag. The efficacy of the developed nanobiocomposite polymer to act as disinfectant material in an environmental media (e.g., sewage wastewater sample) compared to the enriched media (e.g., nutrient Muller Hinton broth) was investigated. From the results obtained, in an environmental media, pMWCNT/CD/TiO_2_-Ag nanobiocomposite polymer can alter the bacteria’s metabolic process by inhibiting the growth and killing the bacteria, whereas, in enriched media, the bacteria’s growth was retarded.

## 1. Introduction

A nanobiocomposite polymer is classified as a polymer matrix nanocomposite (PMNC) and can be defined as a material with multi phases where one of the phases has nanoscale additives. This nanobiocomposite polymer possesses excellent multifunctional properties resulting from each component’s combination. Additionally, these excellent multifunctional properties are essential for their application in various fields, especially water treatment [1].

Water is essential for life on earth, but its scarcity currently restricts the access to clean water due to climate change and population growth, as well as its contamination by sewage, domestic wastes, agricultural pollutants (e.g., pesticides), and various industrial activities (e.g., the use of chemicals containing heavy metal ions, dyes, and organic compounds). These water contaminants have been classified into three main classes, such as organic (dyes), inorganic (e.g., metal ions), and pathogenic microorganisms (e.g., *E. coli*) [2]. The removal of these three classes of water contaminants is a worldwide challenge, and these pollutants have been demonstrated to affect human health and aquatic biota indirectly and directly. For instance, heavy metal ions pollutants (e.g., cobalt ions (Co^2+^)) and organic pollutants (e.g., trichloroethylene (TCE)) have been reported as toxic and carcinogenic; they can also produce bad taste, color, or odor problems. On the other hand, pathogenic microorganism’s pollutants present in water are the source of waterborne diseases [2,3].

In this regard, several water treatment technologies such as membrane filtration, sedimentation, coagulation, chemical oxidation, and chemical precipitation have been developed mainly based on removing these organic and heavy metal pollutants [2,4] while forgetting that the pathogenic microorganism pollutants also coexist in wastewater. Moreover, these methods’ operation was expensive, not very useful for removing pollutants, and produces toxic by-products in the form of sludge. Therefore, to overcome these limitations, the adsorption processes have been adopted; they appear to be the most used and preferred methods. This is because it is not costly; it is simple to design and operate; it can also recycle the adsorbent material [5,6,7]. However, adsorption methods are not exploited to their full potential because of a few limitations, such as the adsorbent’s surface area and adsorption capacity. The challenge is to design an adsorbent material that can purify water by destroying the pathogenic microorganisms and reducing organic and heavy metals pollutants to ultra-trace levels.

Hence, in this work, developing a multifunctional nanobiocomposite polymer capable of removing organic, inorganic, and microorganisms’ pollutants efficiently and effectively to ultra-low levels from wastewater samples will be of a great interest for water purification. This nanobiocomposite polymer is made from the mixture of phosphorylated multiwalled carbon nanotube (pMWCNT), insoluble cyclodextrin (CD) polyurethane, silver nanoparticles (Ag), and titania dioxide (TiO_2_) to obtain a PMNC (pMWCNT/CD/TiO_2_-Ag). Each component in the developed PMNC has been reported to have an essential role during the water treatment. For example, during the process of sorption, the pollutants are attached to the functional groups present on the pMWCNTs surface or be trapped on the hydrophilic exterior of CD through the formation of covalent bonds or chemical interaction [2,4,8], the TiO_2_ nanocatalysts remove and degrades various organic pollutants (e.g., natural organic matters (NOM)) from water. At the same time, Ag nanoparticles act as antimicrobial agents [9]. Furthermore, from previous work reported on insoluble cyclodextrin (CD) polyurethane nanocomposites their efficacy to act as disinfectant material in an environmental media (e.g., sewage wastewater sample) compared to the enriched media (e.g., nutrient Muller Hinton broth) has not yet been reported. Additionally, Research studies on the removal of TCE, Co^2+^, and bacteria using cross-linking polyurethane cyclodextrin nanocomposites are limited.

The present study focused on the removal of heavy metal ions (Co^2+^), chlorinated organic pollutants (TCE), and bacteria (*Bacillus subtilis* (*B. subtilis*) and *Escherichia coli* (*E. coli*)) from synthetic and real wastewater samples using a novel nanobiocomposite polymer (pMWCNT/CD/TiO_2_-Ag) as an adsorbent and disinfecting material.

## 2. Results and Discussion

**Figure 1:**
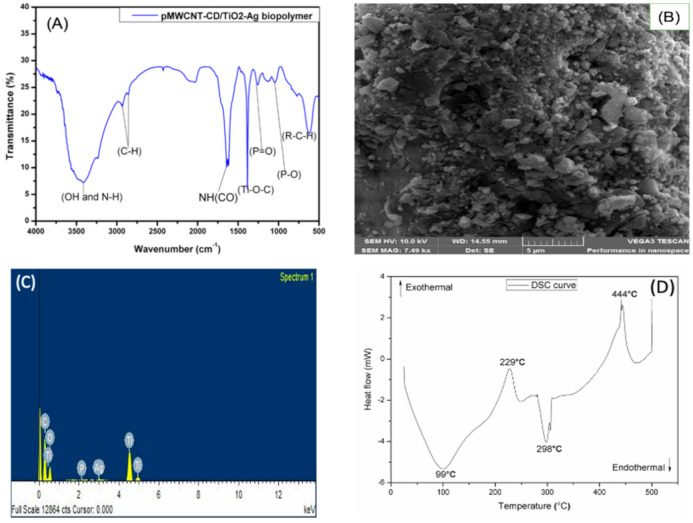
(**A**) FTIR spectrum, (**B**) SEM picture, (**C**) EDX spectrum, and (**D**) DSC curve of the developed nanobiocomposite polymer (pMWCNT/CD/TiO_2_-Ag).

The synthesized nanobiocomposite polymer physically appears as a light grey powder. The BET surface area of the new material was 352.55 m^2^/g. The functional groups (OH, N-H, P=O, P-O, C-H, NH(CO), and Ti-O-C) present on the surface of the prepared pMWCNT/CD/TiO_2_-Ag were confirmed by FTIR spectroscopy (Figure 1A). Among these functional groups, the existence of the carbamate linkage (NH(CO)) distinctive peak at around 1645 cm^−1^ confirms that the polymerization reaction was successful. DSC was used to evaluate the thermal stability of the pMWCNT/CD/TiO_2_-Ag adsorbent. The developed adsorbent proved to be stable and decomposed only at temperatures above 400 °C (Figure 1D). One can also depict that the synthesized pMWCNT/CD/TiO_2_-Ag has a DSC curve (Figure 1D) comparable to that of a semi-crystalline polymer and polymorph material (i.e., it exists in more than one crystal forms). This observation was further confirmed by X-ray diffraction (XRD) spectroscopy, as detailed in our previous study [10,11]. From the SEM picture (Figure 1B), the developed nanobiocomposite polymer has a rough surface, and it seems to be an aggregation of granular particles (or cluster). The EDX result (Figure 1C) shows the different elemental composition of synthesized pMWCNT/CD/TiO_2_-Ag. Additionally, more details on developed nanobiocomposite polymer synthesis and characterization results are reported in our previous work [10,11].

The sorption of Co^2+^ and TCE, respectively, onto pMWCNT/CD/TiO_2_-Ag was dependent on the adsorbent dose, contact time, temperature, initial concentration, and solution pH. The respective removal percentage removal for each pollutant and the sorption capacity [4] were calculated using the Equations (1) and (2) shown below:

% Removal = [(C_o_ − C_e_)/C_o_] × 100 (1)

q_e_ = [(C_o_ − C_e_)/m] × V (2)

where C_o_ is pollutant initial concentration, C_e_ is final concentration at a specific contact time, q_e_ is sorption capacity, m is adsorbent mass used, and V is used volume of the metal ion pollutant solution.

The effects of solution pH and adsorbent dosage are necessary studies for sorption experiments. The study of these factors helps establish the optimum pH and adsorbent dosage; that is, the pH and adsorbent dosage at which the highest removal percentage or capacity is attained [12]. As a result, pH values of 7.5 and 2 were chosen as optimum pH to remove Co^2+^ metal ions and TCE pollutants, respectively, while the optimum adsorbent dosage for removing TCE and Co^2+^ were 0.0005 g and 0.1 g, respectively. These optimum values obtained were then used to investigate the effect of contact time and initial concentration.

**Figure 2:**
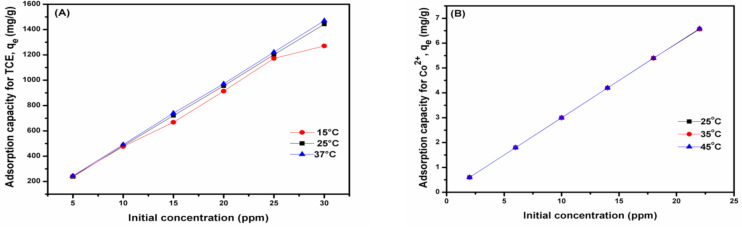
Effect of initial concentration at different temperatures on the adsorption of TCE (**A**) and Co^2+^ (**B**). Conditions: m (adsorbent dose) = 0.0005 g (for TCE) and 0.1 g (for Co^2+^), time = 24 h, pH (TCE) = 2; pH (Co^2+^) = 7.5, V = 30 mL, and speed = 200 rpm.

**Figure 3:**
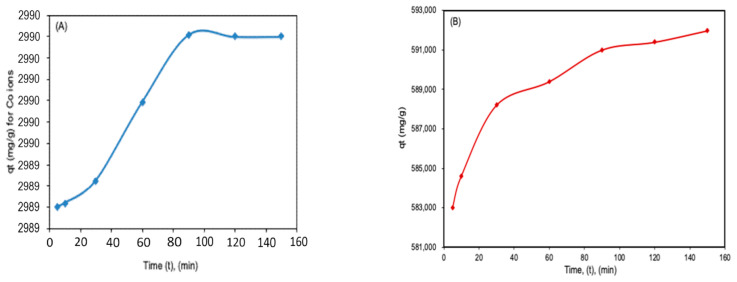
Effect of contact time on the adsorption of Co^2+^ (**A**) and TCE (**B**). Conditions: C_o_ = 10 ppm, m (adsorbent dose) = 0.0005 g (for TCE) and 0.1 g (for Co^2+^), time = 24 h, pH (TCE) = 2; pH (Co^2+^) = 7.5, V = 30 mL, and temperature = 25 °C and speed = 200 rpm.

From the effect of initial concentration, a direct relationship between the initial concentration of pollutants and the sorption capacity was observed (Figure 2), with a maximum capacity of about 1440 mg/g for TCE (Figure 2A) and 6.5 mg/g for the Co^2+^ (Figure 2B) being attained at 30 ppm initial metal concentration. Thus, the prepared nanobiocomposite polymer demonstrated a greater sorption capacity for TCE compared to Co^2+^. It was also noted that the sorption capacity increases with an increase in the pollutants’ initial concentration at all temperatures. Additionally, the effect of contact time was also evaluated; Figure 3A,B show that as the contact time increases, the sorption capacity also increases up to a point where it reaches the plateau whereby no further increases in capacity were observed. The initial increase in capacity with contact time can be explained by the fast rate of adsorbate (Co^2+^ and TCE) diffusion on to adsorbent as the contact time increased. Once the sorption sites on the adsorbent are saturated with the adsorbate species, equilibrium is reached [4,12].

Moreover, to establish the mechanism for the selected contaminant’s sorption onto the pMWCNT/CD/TiO_2_-Ag adsorbent, a set of experimental adsorption isotherm data obtained from laboratory batch tests was fitted with Dubinin–Radushkevich [13] isotherm model and pseudo-second-order (PSO) kinetic model. The kinetic model’s utilization helps to understand better adsorbent’s performance in the sorption process for future applications in the pilot plant [5,14]. The calculated isotherm and kinetic parameters for each of the models are reported in Table 1. The parameters evaluated and listed in Table 1 indicate that the Dubinin–Radushkevich model best explains the sorption mechanism through a multilayer physical adsorption process. In contrast, the PSO kinetic model can be considered as an appropriate model to define Co^2+^ and TCE’s sorption rate on the synthesized nanobiocomposite polymer.

The developed adsorbent’s ability to remove both Co^2+^ and TCE from the real wastewater (mine effluent wastewater sample) was also evaluated. The results obtained are presented in Table 2. From Table 2, it can be depicted that the capacity (q_e_) of pMWCNT/CD/TiO_2_-Ag to remove Co^2+^ was low, which can be explained by the presence of other competing ions in the wastewater sample. In contrast, the synthesized nanobiocomposite polymer’s sorption capacity was very high for TCE removal, which means that the synthesized nanobiocomposite polymer is very efficient for TCE adsorption from the wastewater sample.

The efficacy of the developed nanobiocomposite polymer to act as disinfectant material in an environmental media (e.g., sewage wastewater sample) compared to the enriched media (e.g., nutrient Muller Hinton broth) was investigated. For this study, the collected sewage water as nutrient media for the selected model bacteria was first sterilized. The tests were performed in a sterilized laminar airflow following the standard procedures of antimicrobial susceptibility testing to determine the MIC and MBC [15,16]. The results obtained are summarized in Table 3. These results show that *B. substilis* was more susceptible to pMWCNT/CD/TiO_2_-Ag with the MIC value of 875 µg/mL. Moreover, one can depict a bactericidal activity of pMWCNT/CD/TiO_2_-Ag at a higher concentration using the sewage water as nutrient media.

On the other hand, a bacteriostatic activity at a low concentration of pMWCNT/CD/TiO_2_-Ag was observed in an enriched nutrient Muller Hinton broth media. These results mean that, in enriched media, pMWCNT/CD/TiO_2_-Ag nanobiocomposite polymer can only alter the bacteria’s metabolic process and retard the growth. In contrast, in an environmental condition, pMWCNT/CD/TiO_2_-Ag nanobiocomposite polymer can alter the bacteria’s metabolic process by inhibiting the growth and killing the bacteria. According to the literature, these antibacterial activities observed can be explained by the interactions between the selected bacteria strains and the functional groups existing on the prepared nanobiocomposite polymer’s surface. These interactions formed (i.e., Van der Waals forces, electrostatic interactions) will then produce reactive oxygen species responsible for oxidation stress resulting in a disruption of the bacteria cell wall [17].

## 3. Materials and Methods

The materials and chemicals obtained were utilized as received except the materials used in antimicrobial tests which were first sterilized. Silver nitrate (99.8%) was obtained from Rochelle Chemicals, Johannesburg, South Africa (SA). MWCNTs (purity > 90%), cobalt(II)nitrate hexahydrate (ACS reagent 98%), hexamethylene diisocyanate (HMDI, 98.0%), trichloroethylene (ACS reagent, 99%), N,N dimethylformamide (DMF, 99.9%), 6-amino-hexanol (97%), diethyl(alpha-aminobenzyl)-phosphonate hydrochloride (97%), oxalyl chloride, 2-propanol, titanium tetraisopropoxide (TTIP), and β-CDs (97%) were purchased from Sigma-Aldrich, SA. Nutrient agar, 96 wells micro plates, Petri dishes were obtained from Lasec (Pty) Ltd., SA. Nutrient Muller Hilton Broth and dimethyl sulfoxide (DMSO) were purchased from Sigma-Aldrich, SA. The microorganism strains (*B. subtilis* (ATCC19659) and *E. coli* (ATCC25922)), were bought from Davis Diagnostic, SA. The mine effluent wastewater sample (from a mining area in Okney, North West Province, South Africa) and the sewage wastewater sample (from sewerage wastewater treatment plant at Pretoria, South Africa) were obtained and store in the fridge.

Step 1: The pristine MWCNT was first functionalized by acid oxidation using a mixture of sulfuric and nitric acid (ratio 3:1 respectively). The oxidized MWCNTs obtained were dispersed in DMF (50 mL) and chlorinated using oxalyl chloride (3.35 mL). The reaction mixture was allowed to proceed overnight under an inert atmosphere. Then, the following day, diethyl(alpha-aminobenzyl)-phosphonate (1 g) and 6-amino-hexanol (0.25 g) were added to the reaction mixture to phosphorylate the MWCNTs. The reaction was allowed to continue while stirring at 100 °C for five days. At the end of the reaction, the mixture solution was filtered, washed with ethanol and DMF, and dried at 80 °C for 24 h to produced phosphorylated MWCNTs (pMWCNTs) [10].

Step 2: The cross-linking of CD (1 g) with pMWCNTs (0.05 g in 50 mL DMF) was accomplished via polymerization using HMDI (6 mL) as the cross-linking agent. The reaction was performed under an inert atmosphere at 75 °C for 24 h. After 24 h, the product obtained was precipitated in excess of acetone, filtered, and dried at 80 °C to obtain pMWCNT/CD. Then, the pMWCNT/CD composite polymer was doped with Ag and TiO_2_ nanoparticles through a sol-gel method to obtain a nanobiocomposite polymer, pMWCNT/CD/TiO_2_-Ag [10].

Step 3: The prepared nanobiocomposite polymer, pMWCNT/CD/TiO_2_-Ag, was characterized using techniques such as differential scanning calorimetry (DSC), Fourier-transform infrared (FTIR), Brunauer–Emmett–Teller (BET) method, and scanning electron microscopy-energy dispersive X-ray (SEM-EDX) spectroscopy. DSC analysis was performed using 5 mg of the polymer sample, which was heated from 25 °C to 500 °C at 10 °C/min under nitrogen atmosphere (flow rate 25 mL/min). FTIR spectroscopy analysis was conducted using a mechanical press to obtain a transparent pellet of the polymer sample (made by mixing 1 part of pMWCNT/CD/TiO_2_-Ag with 99-part potassium bromide). The FTIR spectrum obtained was scanned from 4000 to 400 cm^−1^ at a resolution of 4 cm^−1^ with 16 scans. For the BET analysis, the sample was degassed at 100 °C under a nitrogen atmosphere for four hours. The polymer sample used for SEM-EDS analysis was prepared by gold coating the carbon tape containing few mg of the synthesized polymer sample.

Step 4: The synthesized adsorbent material was tested for its capacity to efficiently and effectively remove Co^2+^ and TCE from wastewater samples by batch adsorption study. The preparation of stock solutions (1000 ppm) and model solutions (2–30 ppm) for each pollutant as reported in our previous studies [6,18]. The batch adsorption experiments were conducted at room temperature as follow: 30 mL of the prepared model pollutant solutions were added in respective bottles containing a certain amount of the adsorbent dose (depending on the parameters under investigation), and the pH was adjusted to the value of 7.5 (for Co^2+^) and 2 for TCE. Then, bottles were agitated using a thermostatic platform shaker at 200 rpm. After adsorption, the samples were filtered using 0.45 µm PTFE syringe membrane filters. The filtrates were then analyzed, respectively, by ICP-OES and GC-MS to quantify the remaining amount of Co^2+^ and TCE in the filtrates obtained. The parameters such as pH, adsorbent dose, initial concentrations, and contact time were investigated to determine the optimum conditions for Co^2+^ and TCE’s adsorption from the mine effluent wastewater sample by the developed pMWCNT/CD/TiO_2_-Ag. The adsorption behavior and potential of pMWCNT/CD/TiO_2_-Ag were determined through isotherm and kinetics studies.

Step 5: The disinfection of the sewage wastewater sample collected was assessed based on the antimicrobial susceptibility testing methods by determining the minimal inhibition concentration (MIC) and minimal bactericidal concentration (MBC). The ability of the developed pMWCNT/CD/TiO_2_-Ag to act as a disinfecting agent in an enriched nutrient Muller Hinton broth media compared to an environmental media (e.g., sewage wastewater) was also evaluated.

## 4. Conclusions

The developed nanobiocomposite polymer (pMWCNT/CD/TiO_2_-Ag) physically appears as a light grey powder and has a rough surface. It has an excellent capacity to be used as a filter or disinfectant material for wastewater purification. The data acquired helped to confirm the capability of pMWCNT/CD/TiO_2_-Ag to remove organic, inorganic, and microorganism’s pollutants from contaminated water samples. The Dubinin–Radushkevich model best explains Co^2+^ and TCE’s sorption mechanism through a multilayer physical adsorption process onto pMWCNT/CD/TiO_2_-Ag, while the PSO kinetic model is an appropriate model to define the adsorption rate of Co^2+^ and TCE. Besides, nanobiocomposite polymer was designed to be as cost-effective as possible that is, it can be regenerated and re-used.

## Figures and Tables

**Table 1:** Adsorption models and evaluated parameters.

Models	Parameters	Removal of Co^2+^	Removal of TCE
Dubinin–Radushkevish (D-R) lnq_e_ = ln(q_m_) − (Kad) E = [1/2√BDR] BDR = Kad ε = RTln [1 + 1/Ce] R = 0.008314 KJ/mol/K	q_m_ (mg/g)	1.9 × 10^15^	2028.395
	K_ad_ (mol^2^/KJ^2^)	1.139	0.123
	E (kJ/mol)	0.663	2.0162
	R^2^	0.978	0.972
Pseudo-second-order	q_e_ (exp.) (mg/g)	2.990	591
	q_e_ (cal.) (mg/g)	2.994	591.72
	K_2_ (g/mg/min)	111.556	0.0105
	R^2^	1	1

**Table 2:** Mine effluent wastewater treated using the developed nanobiocomposite polymer.

Pollutants	Concentration before Treatment (ppm)	Concentration after Treatment (ppm)	Removal Capacity (q_e_) (mg/g)	% Removal
Co^2+^	10	0.495	2.85	95.1
TCE	5	0.220	287	95.6

**Table 3:** Disinfecting ability of the synthesized pMWCNT/CD/TiO_2_-Ag based on the obtained MIC and MBC.

Bacteria Strains	Enriched Nutrient Muller Hinton Broth Media		Environmental Media	
	MIC (µg/mL)	MBC (µg/mL)	MIC (µg/mL)	MBC (µg/mL)
*E. coli*	13.67	13.67 (bacteriostatic)	1750	3500 (bactericidal)
*B. substilis*	875	875 (bacteriostatic)	1750	7000 (bactericidal)

## Data Availability

Data are available from the authors.

## Sample Availability

Samples of the compounds are available from the authors.
